# A Review of the Clinical and Therapeutic Implications of Neuropathic Pain

**DOI:** 10.3390/biomedicines9091239

**Published:** 2021-09-16

**Authors:** Eleonora Balzani, Andrea Fanelli, Valentina Malafoglia, Michael Tenti, Sara Ilari, Annette Corraro, Carolina Muscoli, William Raffaeli

**Affiliations:** 1 Department of Medicine and Surgery, University of Turin, 10124 Torino, Italy; 2 Anesthesia, Intensive Care and Pain Therapy Unit, Department of Emergency and Urgency, Istituto ad Alta Specializzazione Policlinico di Monza, Via Amati 111, 20900 Monza, Italy; andre.fanelli@gmail.com; 3 ISAL Foundation Institute for Research on Pain, 47922 Rimini, Italy; valentinamalafoglia@yahoo.it (V.M.); tenti1990@gmail.com (M.T.); wraffaeli@yahoo.it (W.R.); 4 Department of Health Science, Institute of Research for Food Safety & Health (IRC-FSH), University “Magna Graecia” of Catanzaro, Viale Europa, Loc. Germaneto, 88100 Catanzaro, Italy; sara.ilari@hotmail.it (S.I.).; muscoli@unicz.it (C.M.); 5 Faculty of Social and Behavioural Sciences, Leiden University, 2311 Leiden, The Netherlands; annette.corraro@gmail.com

**Keywords:** neuropathic pain, pain therapy, tailored therapy

## Abstract

Understanding neuropathic pain presents several challenges, given the various mechanisms underlying its pathophysiological classification and the lack of suitable tools to assess its diagnosis. Furthermore, the response of this pathology to available drugs is still often unpredictable, leaving the treatment of neuropathic pain still questionable. In addition, the rise of personalized treatments further extends the ramified classification of neuropathic pain. While a few authors have focused on neuropathic pain clustering, by analyzing, for example, the presence of specific TRP channels, others have evaluated the presence of alterations in microRNAs to find tailored therapies. Thus, this review aims to synthesize the available evidence on the topic from a clinical perspective and provide a list of current demonstrations on the treatment of this disease.

## 1. Introduction

Neuropathic pain (NP) is a type of pain arising as a direct consequence of a lesion, dysfunction, or disease affecting the somatosensory system [1]. Estimating the prevalence and incidence of NP presents difficulties due to the high number and diversity of diagnostic criteria employed in the clinical practice, according to each specialization [2]. Recently, a questionnaire has been developed by including screening tools that should help in the assessment of NP. By using these tools, the prevalence of NP has been estimated at around 7–10% [3]. Moreover, the frequency of chronic NP is higher in women (8%) than in men (5.7%) and is more common in patients over 50-year-old (8.9%) than under 50-year-old (5.6%). In addition, chronic NP mostly involves the lower and upper limbs, lumbar spine, and the neck [4]. NP includes several heterogeneous pathologies characterized by the presence of a persistent and/or recurrent state of pain, either associated or not with alterations of somatic-sensory perceptions. These alterations might spread around a single nerve or nerve plexuses, around the spinal-cortical areas with qualitative pain features that might vary based on the specificity of the pathological conditions (for example the trigeminal neuralgia, painful radiculopathy, diabetic neuropathy, HIV infection, leprosy, or within a complex disease such as post-herpetic neuralgia), as post-herpetic neuralgia, and pain from peripheral nerve damage, producing a chronic pain regional syndrome type I or type II or for a central nervous system damage as central post-stroke pain or spinal diseases [5].

NP pathophysiology is extremely complex, thus justifying the absence of optimal therapy. The efficacy of the treatments employed to manage NP is often variable, leading to a continuous replacement of drugs of even more uncertain efficacy [6,7]. Consequently, the purpose of this review is to analyze the underlying pathophysiologic mechanism of NP, to check the current diagnostic tools, outline the pharmacologic and non-pharmacologic treatments available for NP, and propose future perspectives for the evaluation and treatment of NP.

## 2. Pathophysiologic Mechanisms Underlying Neuropathic Pain

**Figure 1:**
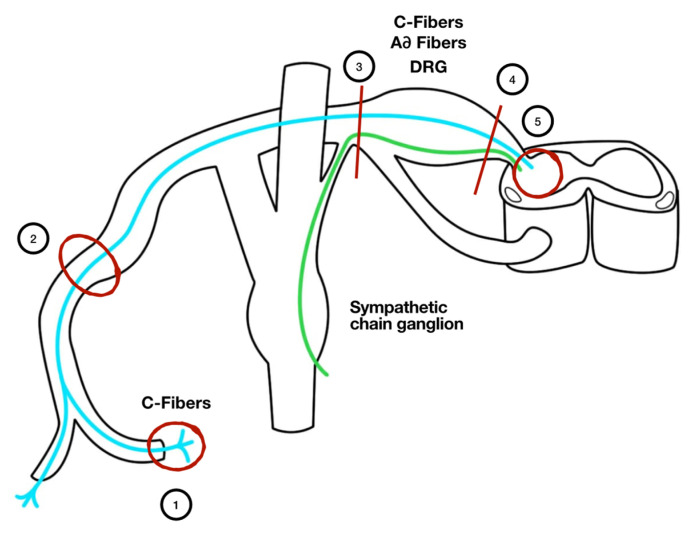
Different anatomical localizations originating from different types of neuropathic pain. 1. Receptor hyperexcitability, mediated by a dysfunction of C-fibers. 2. Demyelination, or alteration of the myelin sheath. 3. NP from ganglion distal lesion due to massive depolarization of a nerve section, changes in axoplasmic transport which may be caused by amputation, hyperexcitability of ganglion cells (derived from neuroma), production of ephaptic transmission. 4. Degeneration of C-fibers and central sprouting of terminals Aß fiber (lamina II). This alteration occurs in the posterior horn lamina II of the spinal cord. 5. Central NP. Small fiber neuropathy and central hyperexcitability pain enhancement are not shown in the figure. DRG: dorsal root ganglion.

The mechanisms underlying NP are numerous, and not fully understood yet. To better explain the underlying pathophysiology of NP, we categorize it according to the different anatomical sites in which the neuronal dysfunction develops (pain generator): NP from nociceptor hyperexcitability, NP from myelin sheath alterations, NP from lesion distal to the ganglion, NP from lesion proximal to the ganglion, NP from central nervous system areas, central NP mainly caused from stroke or spinal cord injury [8]. All the mechanisms described are summarized in Figure 1.

Receptor hyperexcitability NP is caused by an increase of sodium channels that destabilizes the cell membrane. In some individuals, the causes of transient hyperexcitability persist over time, which have been partially explained by the co-participation of TRP channels and microglia activation. This type of damage is associated with a burning sensation, static and thermal allodynia caused by heat (C-fiber mediated), and skin warmer than the normal which gets worse when exposed to the heat and improves when exposed to cold. In this case, there are not sensory deficits as the disruption of the nerve fiber is absent. When the mechanisms of sodium channels are activated, there might be an increase in alpha-adrenergic logans in nociceptors connected to C-fibers which reinforce the pain sensation. Although new studies suggest a correlation between the activated TRP channel and the trigger, the mechanism of hyperexcitability is still not fully comprehended.

Demyelination NP might be caused by hypermyelination or demyelination of Aδ-fiber, causing sensorial, and motorial impairments. Hypermyelination leads to an increased duration of the action potential. If the action potential lasts long, it might excite the axon tract either in an orthodromic or antidromic way [9]. Demyelination causes a delay in nerve transmission resulting in increased sodium channels by compensation. Successively, the progressive increase of sodium channels along the axon causes pathological hyperexcitability of the neuron.

Neuropathic pain due to ganglion distal lesion is a type of lesion affecting all the sensory fibers (Aδ, Aß, C-fibers), efferent motor, and sympathetic fibers. Clinically the presence of hypoesthesia, hypo-analgesia, motor deficits, and alteration in reflexes can be observed.

A proximal lesion to the ganglion leads to a degeneration of C-fibers with central sprouting of Aß-fibers. It differs slightly from the other causes as it affects the A afferent fibers (which are connected to lamina II and C-fibers), thus allowing this pathway to be activated also by Aß tactile and Aα proprioceptive fibers [10].

Central NP originates from abnormal activity of damaged central neurons [11]. When generated by a non-centra primary lesion, thus the centralization is secondary to the peripheral cause, it is called central hyperexcitability pain enhancement. Therefore, the etiopathogenesis of NP should always be evaluated. Moreover, the central mechanisms involve the central system of glutamate, already recognized in contributing to the phenomenon of wind-up [2]. In addition, the descending pathways starting from the rostral ventromedial medulla facilitate the maintenance of pain. New studies are currently recognizing further possible areas by which NP might be supported or areas of activation during its chronicization.

Areas of activation motivated in part association to anxiety, depression, and sucrose preference [12]. It is also important to mention small fiber neuropathy, as it constitutes a separate cause of peripheral nerve pathology leading to NP. It consists of a progressive disfunction of C-fibers and Aδ fibers leading either to disorders of temperature and pain sensation, and autonomic disorders [13].

Small fiber neuropathy should also be mentioned as it constitutes a separate cause of peripheral nerve pathology leading to NP. It consists of a progressive disfunction of C-fibers and Aδ fibers leading to not only disorders of temperature and pain sensation, but also autonomic disorders [14].

## 3. Diagnosing Neuropathic Pain

NP is more difficult to assess than nociceptive NP, due to the intensity of the stimulus and its qualitative and subjective characteristics. In Table 1, we report the tools used in assessing neuropathic pain and the context in which they are used.

The Douleur Neuropathique 4 (DN4) questionnaire and the Leeds Assessment of Neuropathic Symptoms and Signs (LANSS) pain scale are the main questionnaires to assess NP, either for the high sensitivity and specificity and for the clinical medical examination they underline to directly explore all types of sensitivity.

New ongoing studies are searching for techniques that can quantitatively and qualitatively objectify NP, such as functional magnetic resonance imaging, and PET scanning using translocator protein-binding radioligands [12]. From the perspective of NP objectification, microneurography could be a useful diagnostic tool and the same for electroencephalography used as a biomarker of NP [12].

Finally, we should point out that anxiety, depression, sleep disturbances, and poor quality of life contribute to the genesis and maintenance of NP. Special tests for these variables, such as the Short Form-36 Quality of Life Questionnaire, should be evaluated in this context [6,20].

The physical examination evaluates the presence of hyperalgesia, allodynia, and hypoesthesia through an assessment using flexor reflexes, peripheral magnetic resonance imaging, quantitative sensory tests, neurophysiological tests such as laser-evoked potentials, microneurography, skin punch biopsy, evaluation by confocal corneal microscopy, or intraepidermal nerve fiber density [5].

An accurate clinical examination is essential at the time of diagnosis, as pronounced mechanical and dynamic allodynia and thermal sensory loss (with pressure and pain hyperalgesia) could confirm small fiber damage. Differently, the presence of paradoxical heat sensation could reveal the involvement of larger fibers. Thermal hyperalgesia should also be investigated, either caused by heat or cold since it could suggest ectopic activity of nociceptors.

## 4. Treatment of Neuropathic Pain

Recently, an algorithm based on international guidelines has been published suggesting the necessary steps to treat NP [21]. After formulating a diagnosis of NP, it is essential to promote a functional improvement in the individual’s quality of life, a regularization of sleep-wake rhythm, mood, and social status. This step requires a multidisciplinary team.

The first step consists of medications, such as tricyclic antidepressants, selective serotonin reuptake inhibitors, gabapentinoids, and topical medications such as lidocaine, and capsaicin, or transdermal substances. In this first step, the only clear indications for drug use refer to topical lidocaine indicated in postherpetic neuralgia, and 10% transdermal ketamine in complex regional pain syndrome (CRPS) [22,23]. Efficacy requires at least 4 to 6 weeks to assess, after which the second line of treatment can be used.

The second step offers two options: using tramadol or tapentadol, or a combination of multiple dressings from the first step. A Cochrane review demonstrated more effectiveness of the gabapentinoid-opioid combination than gabapentinoids alone [24], whereas no benefit emerged with other drug combinations such as duloxetine and pregabalin [25]. Besides, this type of choice increases the side effects of the drugs and limits their tolerability [26]. The indications for tramadol are specific, i.e., acute NP, cancer-related NP, and intermittent exacerbations of NP. The use of tapentadol is conflicting and not yet well understood.

The third step considers three different classes of drugs, despite no clear indication of them [27]. Alternatively, interventional therapy is proposed. Possible interventions include epidural injections (although not very effective in chronic radiculopathy due to herniated lumbar discs [28]), pulsed radiofrequency, radiofrequency denervation with heat-induced nerve ablation (which in our experience, they should both always be preceded by a test block with a local anesthetic), adhesiolysis in failed back surgery syndrome, or radiculopathy [29], sympathetic block in complex regional pain syndrome [30], and lastly the approach with the endoscopic epidurolisys technique as the first interventional step for complex neuropathic pain syndrome, such as failed back surgery syndrome.

Neurostimulation is used only in the fourth step, although it is not considered optimal for certain types of NP, according to NeuPSIG recommendations, such as postherpetic neuropathy (PHN), diabetic peripheral neuropathy (DPN), spinal cord injury, and poststroke pain [29]. Neurostimulation is a mandatory step before initiating chronically given low-dose opioid therapy [21]. Neurostimulation is evolving with increasingly effective techniques such as high-frequency and burst spinal cord stimulation, and dorsal root ganglion stimulation, which seems to decrease pain and have fewer side effects compared to drug therapy [31].

In the fifth step, there are low-dose opioids. First, there is no specific guidance on which type of opioid is more indicated than the other. Second, there is no clear difference from placebo treatment in the context of chronic low back pain [32]. The opioids considered in this context are morphine, oxycodone, methadone, and levorphanol. In our opinion, this is a critical point because these drugs’ mechanism of action is not directed to the nociceptive component, but rather acting by sedating the central cognitive function, and thus inducing compulsive abuse. For this reason, CDC and Canadian guidelines recommend, at this stage, optimizing the nonpharmacological and non-opioid-based therapies [33,34]. Despite the underlying rationale, the efficacy of this type of medication greatly differs by the type of drug and the center performing the treatment, and for this reason, the NeuPSIG recommendations have not been able to address this issue [29]. In our experience, ziconotide has been beneficial in refractory pain with a safe profile [35]. In this step, we suggest considering other types of therapies, such as transcranial direct-current stimulation, and repetitive transcranial magnetic stimulation that has proven to be effective in the setting of NP refractory to all previously listed therapies [36].

Lastly, regarding pharmacotherapy, it should be noted that a combination of drugs is a strategy that has not yet been thoroughly studied, but on which, future research could be structured.

Regarding physical therapy, there are a variety of treatment modalities that can be employed in various conditions, and these strategies should be considered when pharmacotherapy alone is no longer sufficient in the management of NP. Similarly, specific rehabilitation techniques are indicated in specific pathologies, for example, mirror therapy in phantom limb pain, CRPS, and stroke pain, as well as the complementary use of acupuncture for spinal cord injury [37].

When discussing pharmacotherapy in the setting of NP, the clinical trial-proven efficacy of this treatment should always be considered. Normally there is a clinical improvement in pain expressed by approximately two points on the visual analogue scale immediately post-treatment, but only in 49% of cases the patients maintain a reduction of their pain at the three-month follow-up, with an average reduction in pain on the numeric rating scale of 1.3. The same effect was also seen in mood disorders associated with NP [38].

Opioids are similarly associated with short-term NP reduction; Cooper et al. found moderate improvement in NP symptomatology in only 63% of patients. In the same study, an NNT (Number-Needed-to-Treat) of 3.7 (2.6–6.5) was estimated for opioids [39].

It must be specified, however, that in this type of research a preliminary differentiation in the type of pain expressed is not performed. Frequently, an analysis of pain specificity is lacking. On the other hand, a study that evaluates patients’ satisfaction with NP therapy by stratifying subjects according to the type of NP is a survey conducted by Capeda et al. [40]. In this study, the authors collected information from 1502 patients who experienced NP, showing a lower effectiveness in the use of opioids on pain with numbness characteristics, a dissatisfaction expressed in subjects with primarily sharp paroxysmal pain or broad spectrum pain with antidepressant therapies, and a dissatisfaction with opioids in subjects with deafferentation mechanisms experienced [40].

Similarly, invasive therapies such as spinal cord stimulation would seem to be more effective in treating chronic spine and leg pain especially if it results from a failed back surgery syndrome [41,42].

The safety profile of these molecules should also be evaluated. The number needed to harm (NNH) for major adverse drug reactions (ADRs) in the case of high-dose antidepressants has been estimated at 28, while for minor effects it is 9 [43].

In the case of gabapentinoids, the combined NNH, considering 25 randomized controlled trials was 13.9 (11.6–17.4); for opioids, an NNH of 11.7 (8.4–19.3) was estimated [27].

Many times, the safety profile of the molecule should guide the selection of the medication itself, while considering the clinic and the extent of the NP. ADRs are a cause of hospitalizations in patients, especially the elderly [44].

Their incidence rises as the number of chosen molecules increases, thus opioid and gabapentinoid, in spite of greater efficacy in treating NP, also have a greater risk of ADRs than taking these drugs alone [45].

An approach that takes into account renal or hepatic impairment, as well as interaction on isonenzymes such as cytochrome P450 CYP2D6, could certainly increase awareness of the risk of ADRs, preventing major consequences, such as hospitalization [44].

Thus, the risk–benefit ratio for each type of molecule or combination of drugs used in the treatment of NP should always be kept in mind.

## 5. Biomarkers and Neuropathic Pain

Pain is currently defined as self-reported by the patient [46]. Consequently, it becomes difficult to objectify, especially in individuals that cannot effectively communicate pain. Thus, the use of biomarkers represents a central role to facilitate objectification, which is known as well to move therapeutic strategy toward precision medicine. They can enrich the strategy, which can be prognostic, meaning that they can select patients who are likely to be more predisposed to develop NP; or predictive, meaning that they are likely to respond better to an intervention based on a biological mechanism [47].

Recently, a consensus statement regarding the discovery and validation of new biomarkers, involved in the development or the potential use in pain therapy, has been published [48]. From a prognostic point of view, discussing biological biomarkers in NP, there are preclinical biomarkers, such as behavioral, electrophysiological, and other overt signs, and human biomarkers of pain which may be useful in the diagnosis and treatment of NP. Other potentially useful biomarkers with demonstrated clinical efficacy are nerve growth factor for chronic low back pain [49], calcitonin gene-related peptide concentration in migraine [50], and expression of transient receptor potential cation channel, subfamily V (TPRV) [51,52], which has been related to pain states for inflammatory pain.

On the other hand, if we refer to predictive power in response to therapeutic interventions, regarding biomarkers in the context of pain, while microRNAs such as miR-548d might predict a response to intravenous ketamine in complex regional pain syndrome [53]; phosphorylation of TrkA in skin biopsies has shown to have a better response to certain treatments [54]. However, several findings are the result of trials conducted in animal models, or in vitro cells; the few studies on human samples have been instead only conducted on small cohorts of patients.

Therefore, we conducted a systematic review using Pubmed and Embase searching for papers dealing with biomarkers in NP only in human patients. Strings used for the search were: Pubmed (“neuralgia”[MeSH Terms] OR “neuralgia”[All Fields] OR (“neuropathic”[All Fields] AND “pain”[All Fields]) OR “neuropathic pain”[All Fields]) AND (“biomarker s”[All Fields] OR “biomarkers”[MeSH Terms] OR “biomarkers”[All Fields] OR “biomarker”[All Fields]); Embase: (‘neuropathic pain’/exp OR ‘neuropathic pain’ OR (neuropathic AND (“pain”/exp OR pain))) AND (“biomarkers”/exp OR biomarkers). Two authors (AF and EB) screened independently and in duplicate the abstracts of all publications obtained by the search strategies. The literature research retrieved a total of 1344 articles. After deduplication, abstracts of 1120 studies were evaluated. Then, we selected only clinical trials (randomized controlled trials and non-randomized controlled trials) published in English or Italian language which dealt in the title and abstract with biomarkers used in NP. Other exclusion criteria used were the use of animal or in vitro models, on which the studies were conducted, and the presence of genetic syndromes, which, being determined by specific genetic factors, may have completely different pathways leading to the development of NP.

The result that emerged is very heterogeneous (Table 2): multiple biomarkers of different nature were evaluated in different types of samples. The correlations found are not always present, the pathologies considered are quite disparate.

From our brief systematic review, we have established that despite new studies evaluating biomarkers in patients with NP of different causes, it is still difficult to set up a model to assess susceptibility to the development of the pathology or a specific therapeutic intervention. Given the huge variety of available biomarkers involved in the process of neuroinflammation, it is difficult to determine which biomarker to target in future studies.

Nonetheless, biomarkers such as proflogistic cytokines seem to have quite a good correlation with NP development. However, it should also be determined what type of NP is being referred to and what type of sample should be employed. The use of samples that are too difficult to collect, such as cerebral fluid, might fade into the background compared to samples that are easier and less invasive to gather, such as serum, peripheral blood, and saliva.

## 6. Future Perspectives: Molecular Alterations and Tailored Therapy

Due to the several mechanisms underlying NP, new studies have focused on its clustering [72,73] to target therapy based on pathophysiology. On the other hand, other studies focused on the underlying biology of NP to pursue therapies tailored to the molecular issue [74].

Baron et al. presented a three-cohort model to identify subtypes of NP, stratified by damaged nociceptors and survivors of nerve damage [72]. Cluster 1 was described as sensory loss, being clinically determined by loss of tactile, thermic, and painful stimuli, and paradoxical heat sensations [75]. Hypothetically, the underlying pathophysiology is a dying-back type of degeneration in almost all classes of nerve fibers, and the continuous pain seems to be determined by the ectopic activity of damaged nociceptors or CNS neurons [75]. For this type of cluster, Baron et al. recommend a therapy based on antidepressants, and opioids, with lower efficacy for gabapentinoids, and sodium channel blockers [76].

Cluster 2 is described as thermal hyperalgesia and is characterized by moderate conservation of small and large fibers, in association with heat and cold hyperalgesia and dynamic mechanical allodynia [72]. Among these patients, their hyperalgesia depended on peripheral sensitization without sensory loss, which is probably determined by successful skin regeneration and nociceptor re-sensitization, with a clinical profile similar to UV-B burn injury [77]. Thus, in this case, pain becomes chronic due to spontaneous activity in the surviving nociceptors. Therapy with sodium channel blockers, second-line botulinum, topical capsaicin, antidepressants, gabapentinoids, and opioids is indicated in this setting [78,79].

Cluster 3, or mechanical hyperalgesia, is characterized by a loss of sensitivity of small fibers to heat and cold in combination with pressure hyperalgesia, pinprick hyperalgesia, and marked and frequent dynamic mechanical allodynia [72]. In this case, there is hyperalgesia due to centralization [80]. For this type of cluster, it is recommended to use drugs such as gabapentinoids and sodium channel blockers [81,82,83,84].

Successively, another model considers Transient Receptor Potential Channels in the NP [73]. This review conducted by Basso et al. reviews channel-specific dysfunction and the associated pharmacology. Briefly, alterations in TRPV1 result in polymodal and voltage-dependent activation. In addition, sensitization of this channel is associated with the presence of nociceptive molecules such as nerve growth factor (NGF), bradykinin (BK), or prostaglandin E2 (PGE2). This type of alteration is associated with platinum-based chemotherapy. Protease-Activated Receptor 2 (PAR2) seems to be involved in this mechanism. It was indeed observed that blockade of PAR2 or TRPV1 was able to inhibit oxaliplatin-induced neuropathic pain [85]. TRPA1 has been suggested to contribute to noxious cold sensation and mechanical transduction [73]. This channel’s activation is associated with the presence of reactive oxygen species (ROS), toxins and bacterial products, or UV light [73]. Prostaglandins, cyclopentane, and oxidative stress products have been shown to directly trigger TRPA1 [86,87]. In addition, TRPA1 appears to be implicated in cold allodynia caused by nerve injury, and in diabetes-associated peripheral neuropathy [88,89,90,91]. Lastly, TRPM8 plays a dual role in neuropathic pain induced by nerve injury. Its activation has been found to present powerful analgesic properties by alleviating mechanical and cold hyperalgesia in several models of NP [92,93]. In chemotherapy-induced NP, TRPM8 participates in the development of cold hypersensitivity caused by oxaliplatin [94].

In conclusion, noncoding RNAs, namely lncRNAs, circRNAs, and miRNAs, are involved in NP development by many mechanisms [94]. The explanation for this type of phenomenon is that mRNAs and miRNAs appear to be molecules associated with inflammation. Several studies related the expression of miR-138, miR-667, miR-29a, and miR-500 to alterations due to nerve injury, hyperalgesic conditions, and neuroplasticity [95].

The role of exosomes, or extracellular microvesicles involved in intercellular communication, is not negligible in this context. These types of structures are involved in pathologies that determine both inflammatory and NP, namely osteoarthritis, rheumatoid arthritis, inflammatory bowel diseases, neurodegenerative pathologies, complex regional pain syndrome, and peripheral nerve injury [96,97,98,99,100,101]. Regarding NP, exosomes are released and taken up by neurons based on synaptic activity, enabling inter-neuronal communication [102]. A chemokine, specifically Ccl3, would appear to mediate central sensitization in neuropathic pain through Schwann cells, as well as the p75 and the neural cell adhesion molecule (NCAM) exosome proteins [103]. Several other mechanisms of NP are related to the alteration of exosomes, both in mouse and human models [104,105,106].

The role of exosomes is of particular interest given that through the use of these intercellular messengers, anti-inflammatory information is possible, which constitutes an important potential for novel non-invasive therapies in the treatment of NP [107]. Moreover, exosomes play an important diagnostic role. They are indeed involved in processes of synaptic plasticity, neurogenesis, and neuronal differentiation [108]. Alterations in these processes have been received in neurodegenerative diseases and used in their diagnosis as biomarkers on peripheral blood samples [100].

The use of exosomes obtained by mesenchymal stem cells (MSCs) showed an impact in alleviating certain types of chronic pain by transferring miRNAs to target neurons and promoting their growth and survival. Studies have been conducted by transferring glial cell-derived neurotrophic factor (GDNF), fibroblast growth factor-1 (FGF-1), brain-derived neurotrophic factor (BDNF), insulin-like growth factor-1 (IGF-1), and nerve growth factor (NGF) to MSCs-exosomes [109]. In addition, the use of miR-21-5p antagomir, which regulates the expression of miR-21-5p—overexpressed after nerve injury in mice—appears to have an impact on the inflammatory status and onset of neuropathic hypersensitivity [110]. In addition, intrathecal injection of miR-122-5p in murine models has been shown to reduce mechanical allodynia, and thermal hyperalgesia [111].

## 7. Conclusions

To sum up, NP is a broad term that encompasses several types of pain. To date, there are no specific guidelines that indicate a standard therapy to treat it. While the diversification of the various entities constituting NP is still unclear, it appears that it may lead to a personalized therapy that will improve patient outcomes.

## Figures and Tables

**Table 1:** Summary of tools used in neuropathic pain assessment.

Tool	Consistencies	How and When to Use It
Leeds Assessment of Neuropathic Symptoms and Signs (LANSS)	It requires a physical examination. 85% sensitivity and 80% specificity [15].	S-LANSS is the self-reported form. Positive scores on the LANSS or S-LANSS identify patients with pain of predominantly neuropathic origin.
Neuropathic Pain Questionnaire (NPQ)	66% sensitivity and 74% specificity [15].	12 items that include 10 related to sensations or sensory responses, and 2 related to affect.
Douleur Neuropathique 4 Questions	It requires a physical examination. 83% sensitivity and 90% specificity [15].	7 items. A score of 4 out of 10 or more suggests neuropathic pain
painDETECT	Self-reported 85% sensitivity and 80% specificity [15].	9 items. It can be used in neuropathic, nociceptive pain, and low back pain.
Standardised Evaluation of Pain (StEPS)	It requires a physical examination. 92% sensitivity and 97% specificity [16].	It can be used to discriminate between neuropathic (radicular) and non-neuropathic (axial) low back pain.
Neuropathic Pain Scale (NPS)	NA	The NPS quantifies already-diagnosed neuropathic pain. 10 items. A score of more than 4 suggests neuropathic pain
Pain Quality Assessment Scale (PQAS)	Self-reported	20 items. It provides the pain qualities.
ID-Pain	78% sensitivity and 74% specificity [17].	5 sensory descriptor items and 1 item relating joint nociceptive pain.
Neuropathic Pain Symptom Inventory (NPSI)	Self-reported. 91% sensitivity and 70% specificity [18].	Characterize subgroups of neuropathic pain patients.
Neuropathic Pain scale for Postsurgical patients (NeuPPS)	88% sensitivity and 59% specificity [19].	5 items. Measurement of neuropathic pain among postsurgical patients.

**Table 2:** Summary table of biomarkers used for NP.

Author	Biomarker	Sample	Pathology	Evidence
Assi et al. [55]	Thrombospondin 4	Serum	Advanced osteoarthritic neuropathic states	Correlation was demonstrated
Balagué et al. [56]	Keratan sulfate, hyaluronan, and cartilage oligomeric matrix protein	Peripheral blood	Sciatica	No correlation with clinical outcome
Dietz et al. [57]	hsa-miR-223-5p	Plasma	Complex regional pain syndrome	Correlation was demonstrated
Ramanathan et al. [58]	miRNAs	HEK293 cells	Complex regional pain syndrome	Correlation was demonstrated
Ericson et al. [59]	Tumor necrosis factor—related apoptosis inducing ligand, Tumor necrosis factor-beta	Cerebrospinal fluid	Trigeminal neuralgia	Correlation was demonstrated
Hayakawa et al. [60]	Lysophospholipids	Cerebrospinal fluid	Lumbar spinal stenosis	Correlation was demonstrated
Hider et al. [61]	Tumor necrosis factor-alpha, IL-6 and matrix metalloproteinases	Serum	Sciatica	No correlation with clinical outcome
Kallman et al. [62]	Beta-endorphin and substance P	Saliva and salivary-to-plasma quotients	Chronic neuropathic pain patients	No correlation with clinical outcome
Karakulova et al. [63]	Brain-derived neurotrophic factor and vascular endothelial growth factor and TrkB, VEGFR2	Serum	Diabetic polyneuropathy	Correlation with clinical outcome
Kwon et al. [64]	IL-6, IL-8, and MCP-1	Cerebrospinal fluid	Spinal cord injury	Correlation with clinical outcome
Lind et al. [65]	Follistatin, interleukin-1 alpha, and kallikrein-5	Cerebrospinal fluid	Neuropathic pain patients	No correlation with clinical outcome
Radojcic et al. [66]	C1M and IL-6	Serum	End-stage knee osteoarthritis	Correlation with clinical outcome
Ri et al. [67]	Lysophosphatidylcholine and phosphatidylcholine	Serum/plasma	Bortezomib-induced peripheral neuropathy	Correlation with clinical outcome
Ri et al. [68]	Lipid metabolites (1 ether-type lysophosphatidylcholine, 1 PC, 1 ceramide, 1 diacylglycerol, 1 triacylglycerol, and 9 oxFAs)	Serum	Bortezomib-induced peripheral neuropathy	Correlation with clinical outcome
Staats Pires et al. [69]	Major kynurenine and tetrahydrobiopterin pathway metabolites	Serum	Diabetic polyneuropathy	Correlation with clinical outcome
Wang et al. [70]	microRNAs (mir-204-5p, mir-519d-3p, mir-20b-5p, mir-6838-5p)	Peripheral blood sample	Spinal cord injury	Not clear correlation
Xu et al. [71]	Tumor necrosis factor-alpha and interleukin-6	Peripheral blood sample	Spinal cord injury	Correlation with tumor necrosis factor-alpha and clinical outcome

IL-6: interleukin-6; IL-8: interleukin-8; MCP-1: Monocyte chemoattractant protein-1; C1M: type 1 collagen; VEGFR2: Vascular endothelial growth factor receptor 2.

## Data Availability

No new data were created or analyzed in this study. Data sharing is not applicable to this article.
